# Near‐Source Wastewater Surveillance of SARS‐CoV‐2, Influenza A, and Respiratory Syncytial Virus With Spatiotemporal Evaluation of Pepper Mild Mottle Virus as a Fecal Indicator

**DOI:** 10.1002/wer.70414

**Published:** 2026-05-15

**Authors:** Yuzhu Mao, Chunfu Liu, Rushan Abayagunawardena, Kosala Desilva, Patricia Arcellana, Birthe V. Kjellerup

**Affiliations:** ^1^ Department of Civil and Environmental Engineering University of Maryland College Park Maryland USA; ^2^ Public Health Services, Montgomery County Department of Health and Human Services Rockville Maryland USA; ^3^ Inspection Experts, Inc (IEI) Columbia Maryland USA

**Keywords:** influenza A, pepper mild mottle virus (PMMoV), respiratory syncytial virus (RSV), SARS‐CoV‐2, wastewater‐based epidemiology (WBE)

## Abstract

Wastewater‐based epidemiology (WBE) is increasingly transitioning from treatment plant scale monitoring toward community‐scale surveillance to provide higher spatial resolution. However, the influence of monitoring scale on signal stability and normalization performance remains insufficiently characterized. This study evaluated the dynamics of SARS‐CoV‐2, influenza A (IAV), and Respiratory Syncytial Virus (RSV) at five near‐source pumping stations between October 2022 and September 2024. The monitoring sites were selected using the Social Vulnerability Index to ensure representative coverage, and the catchment populations were estimated by integrating census data with physical sewershed boundaries. Wastewater viral concentrations exhibited strong associations with clinical hospitalizations, with maximum Pearson correlations occurring at lead times of 1 day for RSV and 8 days for SARS‐CoV‐2. The performance of Pepper Mild Mottle Virus (PMMoV) as a fecal indicator was target‐specific and scale‐dependent. PMMoV normalization improved predictive correlations and extended the lead time for IAV to 7 days but reduced correlation strength for SARS‐CoV‐2 and RSV, suggesting that raw viral loads may better reflect community shedding for certain pathogens at the neighborhood scale. Comparison with downstream wastewater treatment plant influent revealed substantial signal smoothing at larger spatial scales, whereas upstream pumping stations exhibited higher hydraulic volatility. Small catchments serving fewer than 600 residents showed large signal instability and frequent stagnation. Significant PMMoV seasonality was observed, with consistent winter and spring peaks across sites. Together, these findings indicate that near‐source WBE offers reliable neighborhood‐level surveillance and that the effectiveness of normalization depends on both the viral target and catchment characteristics.

## Summary

- PMMoV exhibited consistent winter–spring seasonality across near‐source catchments.
- Upstream pumping stations showed higher hydraulic volatility than downstream WWTP influent, which displayed strong signal smoothing.
- Small catchments (< 600 residents) produced highly variable signals due to low flow and stagnation.
- PMMoV normalization improved influenza A correlations but reduced SARS‐CoV‐2 and Respiratory Syncytial Virus performance, indicating target‐specific utility.
- Spatiotemporal dynamics and seasonality of viruses in wastewater at neighborhood scale and in larger catchment areas are important to consider for setting up effective and informative surveillance.
- The impact of normalization on the lead times between wastewater measurements and clinical indicators for different viral targets varies depending on the scale of the surveillance area.
- By directly comparing upstream data with downstream WWTP influent from the same region, this work provides a systematic evaluation of scale‐dependent effects on WBE performance.

## Graphical abstract

Wastewater surveillance results showed a 0–8 day early warning compared to clinical data depending on the type of virus that was investigated.

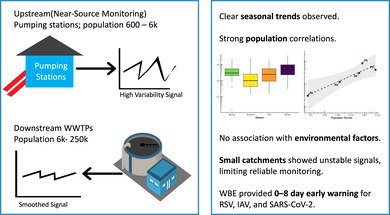

## 1 Introduction

Wastewater‐based epidemiology (WBE) has become an established population‐level surveillance approach for monitoring infectious diseases, mainly seen during the COVID‐19 pandemic. Infected individuals shed viral RNA in feces regardless of symptom status or healthcare access thus wastewater surveillance provides an unbiased and cost‐effective proxy for community infection dynamics (Li, Liu, et al. 2023; Peccia et al. 2020; Singer et al. 2023). Numerous studies have demonstrated strong correlations between SARS‐CoV‐2 concentrations in wastewater and clinical cases, hospitalizations, and test positivity, with wastewater often serving as a leading indicator by several days to weeks (Bibby et al. 2021; Zhu et al. 2021). More recently, WBE has expanded to include other respiratory viruses such as influenza A (IAV) and Respiratory Syncytial Virus (RSV), supporting its role as a multipathogen surveillance platform (Chan and Boehm 2025; Girón‐Guzmán et al. 2024; Zulli et al. 2024). Most WBE programs operate at the scale of wastewater treatment plants (WWTPs), which integrate large populations and provide stable, aggregated signals. Although effective for regional trend monitoring, WWTP‐scale surveillance can obscure localized transmission heterogeneity and delay detection of neighborhood‐level hotspots (Saingam et al. 2024; Schmiege et al. 2024). To address this limitation, near‐source or community‐scale surveillance, such as sampling at upstream pumping stations or manholes, has gained increasing attention. By reducing the distance between the shedding population and the sampling point, upstream monitoring can improve spatial resolution, shorten sewer transit time, and better capture neighborhood‐level transmission dynamics (Saingam et al. 2024; D'Aoust, Towhid, et al. 2021; Nguyen Quoc et al. 2022). However, smaller catchments also introduce challenges, including low and variable flows, longer retention times, and greater susceptibility to stochastic noise, which may compromise signal stability. Ethical challenges must also be considered to preserve anonymity at the individual level, when sampling is moving into neighborhoods.

Normalization is commonly applied in WBE to reduce variability associated with dilution, sample processing, and population size. Pepper Mild Mottle Virus (PMMoV), a plant virus excreted at high concentrations in human feces, is the most widely used fecal indicator (Symonds et al. 2019). PMMoV normalization has been reported to improve correlations between wastewater SARS‐CoV‐2 signals and clinical metrics in some systems (Maal‐Bared et al. 2023). However, an increasing number of studies have shown that the benefits of PMMoV normalization are inconsistent and highly site‐dependent, with improvements observed in only a subset of sewersheds or for specific targets (Maal‐Bared et al. 2023; Goitom et al. 2024; Pappu et al. 2026; Rosengart et al. 2025). Furthermore, PMMoV itself can exhibit spatial and temporal variability driven by dietary patterns, population demographics, and sewer processes, raising questions about its suitability as a universal normalization biomarker (Rosengart et al. 2025; Dhiyebi et al. 2023).

The interaction between monitoring scale and normalization strategy remains poorly resolved. Although large WWTP catchments benefit from hydraulic buffering and signal smoothing, near‐source systems may amplify short‐term fluctuations that normalization cannot adequately correct. At the same time, few studies have systematically evaluated how PMMoV behaves in small, upstream catchments or whether its use improves the predictive performance of wastewater signals for multiple respiratory viruses simultaneously. As WBE transitions toward decentralized and high‐resolution networks, understanding these scale‐dependent effects is critical for designing effective surveillance frameworks.

In this study, we conducted a community‐scale wastewater surveillance program at five near‐source pumping stations in a mid‐Atlantic county, targeting SARS‐CoV‐2, IAV, RSV, and PMMoV. We integrated sewershed mapping with census data to estimate served populations and examined the relationships between wastewater viral concentrations, environmental parameters, and clinical hospitalization data. Specifically, we aimed to (i) characterize the spatiotemporal dynamics and seasonality of PMMoV at the neighborhood scale, (ii) evaluate the influence of catchment population size and hydraulic conditions on signal stability, and (iii) assess whether PMMoV normalization improves correlations and lead times between wastewater measurements and clinical indicators for different viral targets. By directly comparing upstream data with downstream WWTP influent from the same region, this work provides a systematic evaluation of scale‐dependent effects on WBE performance.

## 2 Methods

### 2.1 Site Selection

**FIGURE 1:**
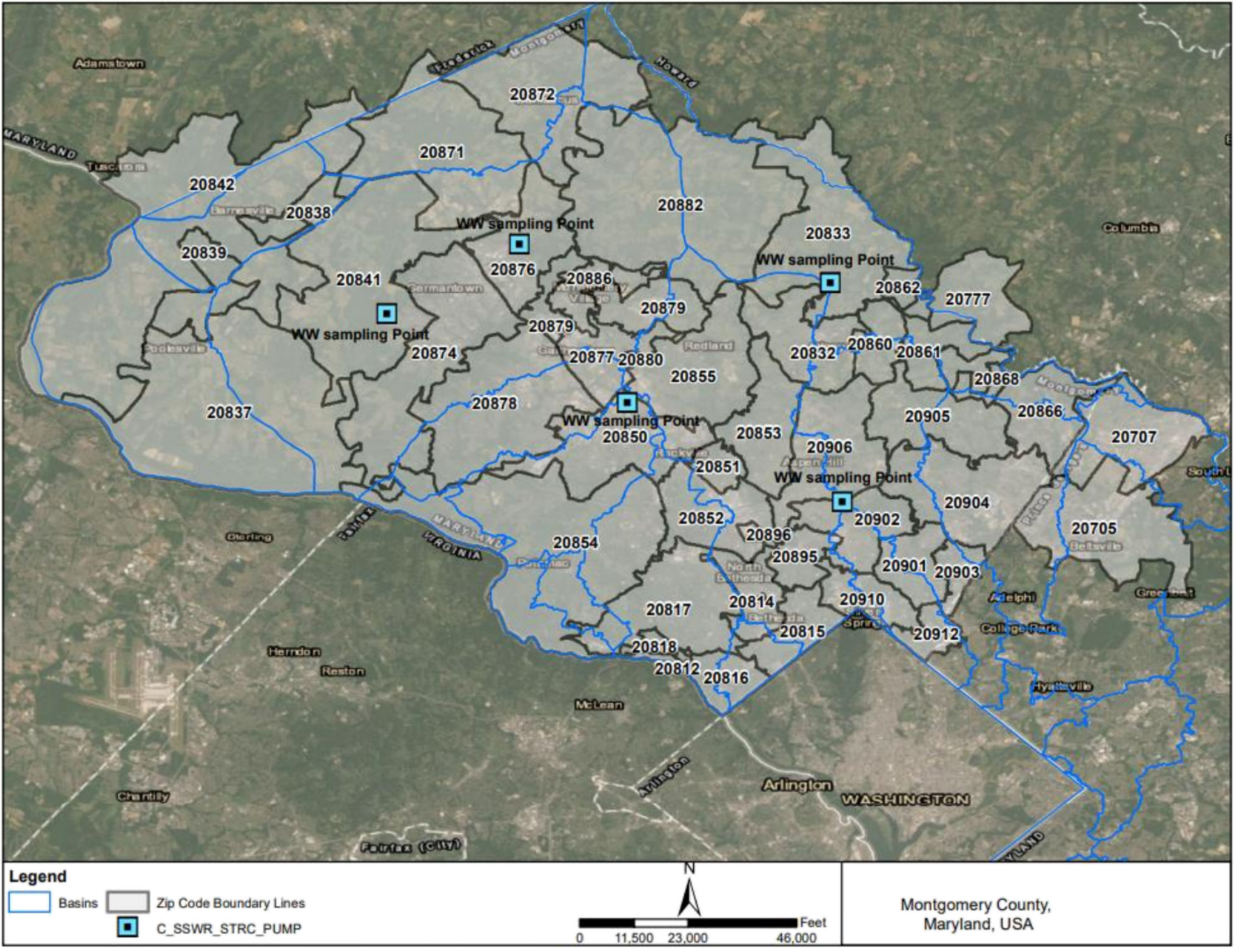
Geographic overview of the study area in Montgomery County, Maryland. The map is showing the wastewater sewershed boundaries (blue outline), locations of near‐source sampling points (blue squares), and corresponding ZIP code boundaries within the study region.

Community‐scale surveillance was conducted at five near‐source pumping stations, designated P1 through P5, in a mid‐Atlantic county. These sites were strategically selected as sentinel nodes using a multistage process that integrated public health indicators, including population density and disease burden, with GIS‐based mapping of sewer basins. This approach resolved discrepancies between administrative zip code boundaries and the physical sewer network, ensuring effective coverage of the target populations. Field validation was performed at each location to confirm logistical suitability for safe and secure autosampler installation and to assess site‐specific flow dynamics. The resulting network represents a diverse cross‐section of the county communities, positioned to capture localized transmission trends through upstream and neighborhood‐level monitoring. Detailed geographic data and site characteristics are provided in Figure 1 and Table 1.

**TABLE 1:** Characteristics of sampling sites and estimated population served.

Sampling sites	Estimated 2023 population	Service area (square miles)	Average flow (MGD)	Note
P1	564	0.53	0.025	Mostly residential area
P2	1704	38	0.09	Shopping centers nearby
P3	3694	48	0.7	Shopping centers nearby
P4	3323	47	0.23	Mostly residential area
P5	5839	29	0.51	Mostly residential area

### 2.2 Sample Collection

Wastewater was collected twice weekly (Tuesdays and Thursdays) from the five designated pumping stations between October 2022 and September 2024. The primary study period for SARS‐CoV‐2, IAV, and RSV dynamics spanned from October 2022 to June 2023, yielding 313 samples distributed as follows: P1 (*n* = 63), P2 (*n* = 63), P3 (*n* = 62), P4 (*n* = 62), and P5 (*n* = 63). Sampling continued through September 2024 to support the longitudinal spatiotemporal analysis of PMMoV concentrations. All events utilized automatic samplers (Great Little Sampler, Teledyne ISCO, NE, USA) to collect 24‐h time‐weighted composite samples into 2.5‐gal plastic bottles. After mixing, 500‐mL aliquots were transferred to sterile high‐density polyethylene bottles, placed on ice, and transported for same‐day (6 h) laboratory processing without freeze–thaw cycles. Wastewater temperature and pH were recorded on‐site during collection.

### 2.3 Wastewater Concentration and RNA Extraction

Two 45‐mL aliquots per sample were transferred to 50‐mL Falcon tubes (Corning, NY, USA) and centrifuged at 3400 ×*g* for 20 min (Allegra X‐22, Beckman‐Coulter, CA, USA) to pellet solids. The supernatant was concentrated to less than 1 mL using Amicon Ultra‐15 Centrifugal Filter Devices (100 kDa cutoff, Millipore, Amsterdam) and adjusted to a final volume of 1 mL with nuclease‐free water (Millipore, MA, USA). RNA was extracted using Quick‐RNA Miniprep kits (Zymo Research, CA, USA). The protocol was modified to process 0.3 mL of concentrate with 0.6 mL of lysis buffer, yielding a 0.1‐mL elution. cDNA synthesis followed the NEB #M0368 protocol (New England Biolabs, MA, USA) with dNTP and random hexamer volumes specified in Table S1.

### 2.4 RT‐qPCR Quantification

Target quantification was performed via two‐step RT‐qPCR using the CFX Connect Real‐Time PCR Detection System (Bio‐Rad, Hercules, CA, USA). Each 20.0‐μL reaction contained 10‐μL TaqMan Fast Advanced Master Mix (ThermoFisher Scientific, MA, USA), 1.0 μL each of forward and reverse primers, 0.5‐μL probe, 4.5‐μL PCR‐grade water, and 3.0 μL of cDNA template. Positive controls for PMMoV, IAV, and RSV utilized 8‐point, tenfold serial dilutions of gBlocks (IDT, IA, USA). For SARS‐CoV‐2 (N1), fivefold serial dilutions of a plasmid control (2019‐nCoV RUO Kit, IDT, IA, USA) were employed. All assays were run in triplicate. Detailed thermocycling conditions, primer and probe sequences, and assay performance characteristics are provided in Table S2 and Table S3.

### 2.5 Population Estimation

Sewershed population metrics were refined by integrating 2020 Census Tract data with the physical catchments of each pumping station using GIS. To ensure high‐resolution accuracy, inhabited land use was delineated by excluding undeveloped regions, such as forests and agricultural areas, from the census tracts. Population densities were subsequently calculated based on these developed zones and applied to the specific spatial extent of each sewershed. The final population for each monitoring site was derived by aggregating these density‐adjusted values across the intersecting census tracts.

### 2.6 Environmental and Clinical Data

Daily COVID‐19 hospital admissions per 100,000 residents were obtained from the County Health and Human Services (HHS) public data portal. Clinical data for IAV and RSV were acquired weekly from the Maryland Department of Health website, aligned with the Morbidity and Mortality Weekly Report (MMWR) schedule. To evaluate environmental and physical influences on viral signals, daily precipitation data were retrieved from local NOAA weather stations, whereas wastewater temperature was recorded directly at each pumping station using on‐site probes during sample collection.

### 2.7 Statistical Analysis

Statistical analyses were conducted using R (version 4.4.1), with significance defined as *p* < 0.05. Spatiotemporal variations in PMMoV concentrations across the five pumping stations were evaluated using Kruskal–Wallis tests followed by Dunn's post hoc analysis for pairwise comparisons. The stability of PMMoV as a fecal indicator at each site was quantified using the Coefficient of Variation (CV %). Additionally, virus mass loading (gc/day) was calculated by multiplying the mean viral concentration (gc/L) by the mean daily flow rate at each site. To ensure comparability between datasets, weekly clinical hospitalization records and semiweekly wastewater concentrations were converted to a daily frequency using linear interpolation. Pathogen concentrations (gc/L) were log_10_ transformed to stabilize variance. Additionally, normalized signals were generated by calculating the ratio of pathogen concentrations to PMMoV concentrations to evaluate whether PMMoV normalization improved the correlation between wastewater signals and clinical trends. The temporal lead–lag relationship between wastewater signals and clinical indicators was quantified using time‐lagged cross‐correlation analysis. Pearson correlation coefficients (*r*) were calculated for lags (𝜏) ranging from −20 to +20 days to identify the offset yielding the maximum correlation for both raw and PMMoV‐normalized data. This optimal lag was then defined as the estimated lead day.

## 3 Results and Discussion

### 3.1 PMMoV Dynamics and Spatiotemporal Stability

**FIGURE 2:**
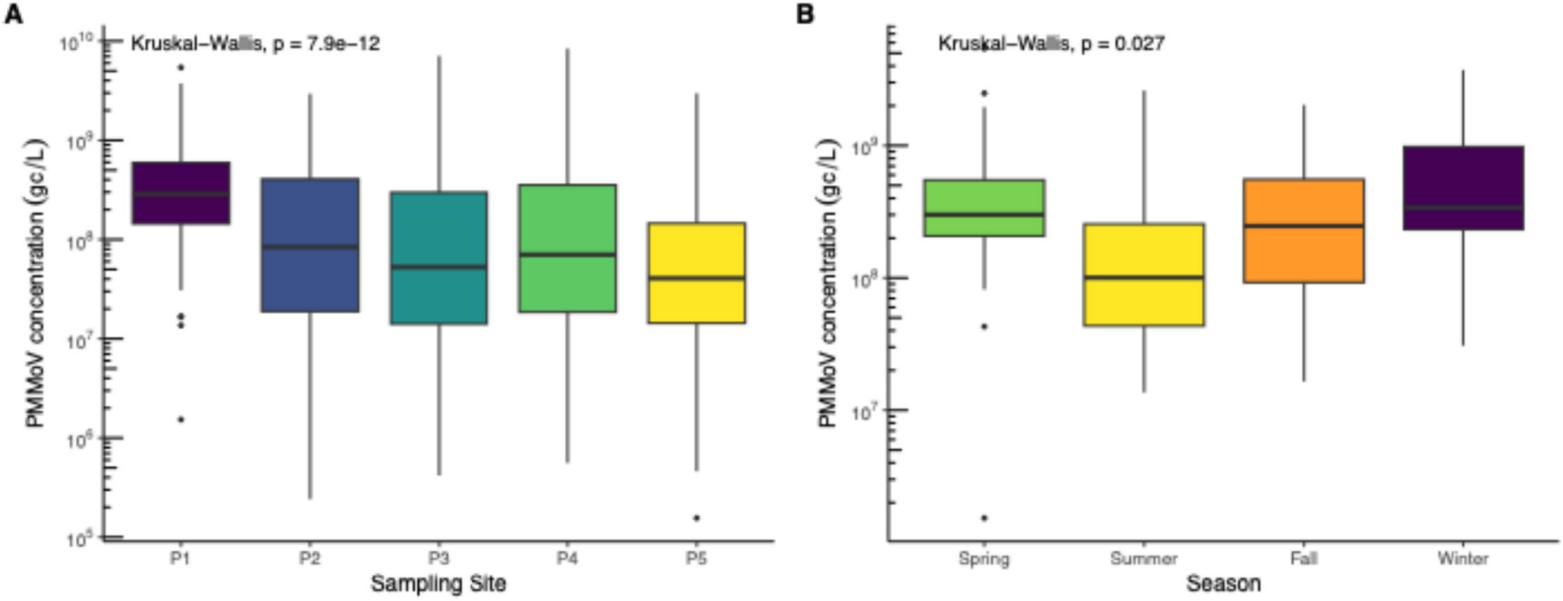
Spatial and seasonal variability of Pepper Mild Mottle Virus (PMMoV) concentrations across near‐source wastewater sites. (A) Boxplots of PMMoV concentrations by sampling site. (B) Seasonal distribution of PMMoV concentrations at Site P1, grouped by winter, spring, summer, and fall.

Spatiotemporal analysis of PMMoV concentrations revealed significant variability across the five near source pumping stations (Kruskal–Wallis, *p* < 0.05). Pairwise comparisons using Dunn's post hoc testing showed that P1 had a distinct profile, with concentrations significantly different from the other monitoring sites (*p.adj* < 0.05 for all comparisons). Specifically, P1 was different from P2 (*Z* = −4.88), P3 (*Z* = −6.38), P4 (*Z* = −5.20), and P5 (*Z* = −7.28). In contrast, differences among P2–P5 were nonsignificant, indicating relative spatial homogeneity across these larger catchments (Figure 2A).

Despite serving the smallest population (*n* = 564), P1 had the highest mean PMMoV concentration (5.75 × 10^8^ gc/L) and the largest standard deviation, resulting in an elevated coefficient of variation. This combination suggests that fecal signal distribution at P1 was strongly influenced by stochastic effects rather than consistent population shedding. The low average flow at this site (0.025 MGD) likely limited hydraulic buffering, allowing short‐term fluctuations in wastewater input to disproportionately affect the measured concentrations.

Beyond hydraulic effects, PMMoV transport is influenced by in‐sewer processes such as adsorption to suspended solids and biofilm interactions. PMMoV is reported to exhibit lower retention prior to biofilm colonization than other fecal indicators, which allow for longer distance transport in the water column compared with organisms such as *E. coli* or *Carjivirus* (Greaves et al. 2026). It is also highly persistent in wastewater, with minimal decay under typical sewer conditions (Chen and Bibby 2025). Together, these factors suggest that variability at near‐source sites is driven more by hydraulics and catchment scale effects than by biological degradation.

To place upstream dynamics in context, PMMoV behavior at pumping stations was compared with influent data from five downstream WWTPs in the same region previously reported by Wartell et al. (2022). From September 2021 to August 2024, 275 influent samples were collected from WWTPs W1–W5 using identical collection and transport protocols. These facilities serve populations ranging from approximately 6‐ to 250‐k residents (Table S4). Although spatial differences were observed among WWTPs, both the magnitude of variation and absolute PMMoV concentrations were substantially lower than those observed at pumping stations. Mean PMMoV concentrations at the WWTPs ranged from 5.17 × 10^7^ to 1.47 × 10^8^ gc/L, indicating signal attenuation and averaging as wastewater aggregated downstream. WWTPs also showed lower CV values, reflecting greater temporal stability (Table S5). These results support a conceptual model in which PMMoV undergoes progressive “smoothing” with increasing catchment size. As wastewater from multiple subcatchments converges, short‐term fluctuations are dampened, producing a more stable normalization baseline than is typically observed at upstream sites.

Seasonal analysis further revealed significant temporal differences in PMMoV concentrations at all five pumping stations (Kruskal–Wallis, *p* < 0.05 for each site). Across locations, PMMoV concentrations were consistently highest during winter and spring and lowest during fall and summer (Figure 2B; Figure S1). Significant seasonal differences were also observed at WWTPs (*p* < 0.05), with the exception of W5 (*p* = 0.525). The drivers of PMMoV seasonality remain uncertain, but the variability may reflect seasonal dietary patterns, produce availability, and regional differences in pepper consumption (Symonds et al. 2019; Paslay and Ali 2025). Prior PMMoV studies report mixed findings, with some observing no seasonal variation (Persson et al. 2025; Nguyen Thanh et al. 2025; Rainey et al. 2023), whereas others document higher PMMoV concentrations in late fall and winter (Dhiyebi et al. 2023) or elevated spring levels (Eifan et al. 2023). Taken together, these results indicate that PMMoV is not temporally constant and that seasonality may differ by region, complicating its interpretation as a universal normalization biomarker. Consistent with Rosengart et al. (2025), much of the observed PMMoV variability appears attributable to geographic location and site‐specific characteristics rather than solely environmental parameters.

### 3.2 Influence of Catchment Population and Environmental Factors

**FIGURE 3:**
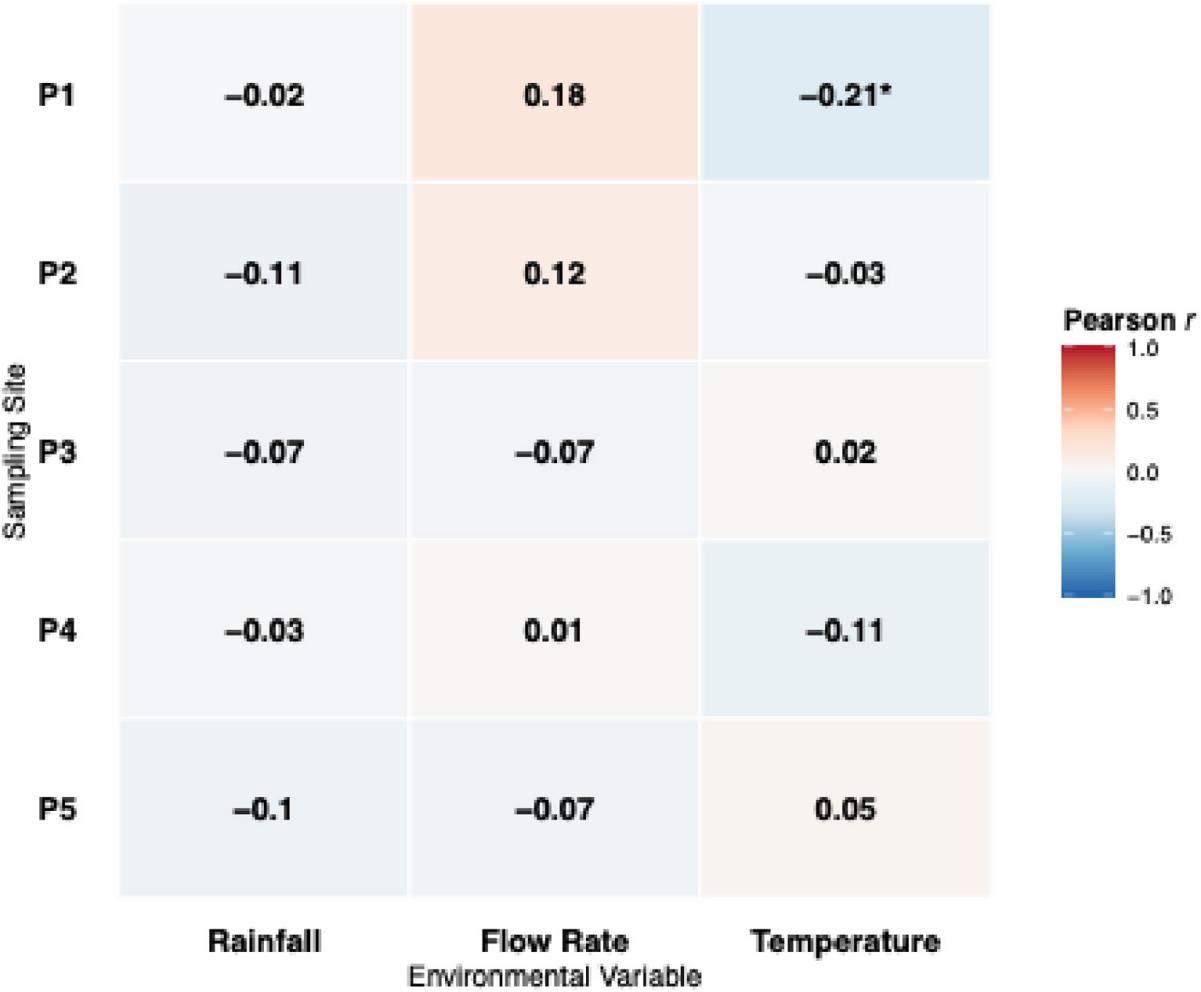
Associations between PMMoV concentrations and environmental variables. Heatmap of Pearson correlation coefficients between PMMoV concentrations and environmental variables. Color intensity indicates the strength and direction of correlations.

Environmental parameters, including wastewater temperature, precipitation, and influent flow, were examined to assess their influence on PMMoV variability. Mean daily flow rates ranged from 0.014 million gallons per day (MGD) at P1 to 0.78 MGD at P3 (Figure S2). Although seasonal temperature patterns and precipitation events were observed, correlation analysis demonstrated that these factors exerted minimal influence on PMMoV concentrations. The only association was a weak negative correlation between precipitation and PMMoV at P1 (*r* = −0.21, *p* < 0.05), whereas all other sites showed no significant environmental relationships (Figure 3).

These findings are consistent with reports that PMMoV concentrations are often weakly related to rainfall or temperature (Maal‐Bared et al. 2023; Pappu et al. 2026; Persson et al. 2025). However, the literature findings remains mixed, with some studies suggesting that storm‐driven scouring can increase PMMoV concentrations (Dhiyebi et al. 2023), whereas others report higher levels during dry weather or rising wastewater temperatures (Maal‐Bared et al. 2023). Another group of studies observed dilution during large precipitation events in combined sewer systems (Rosengart et al. 2025). Such discrepancies likely reflect differences in‐sewer system design, hydrology, and sample processing rather than universal PMMoV behavior.

**FIGURE 4:**
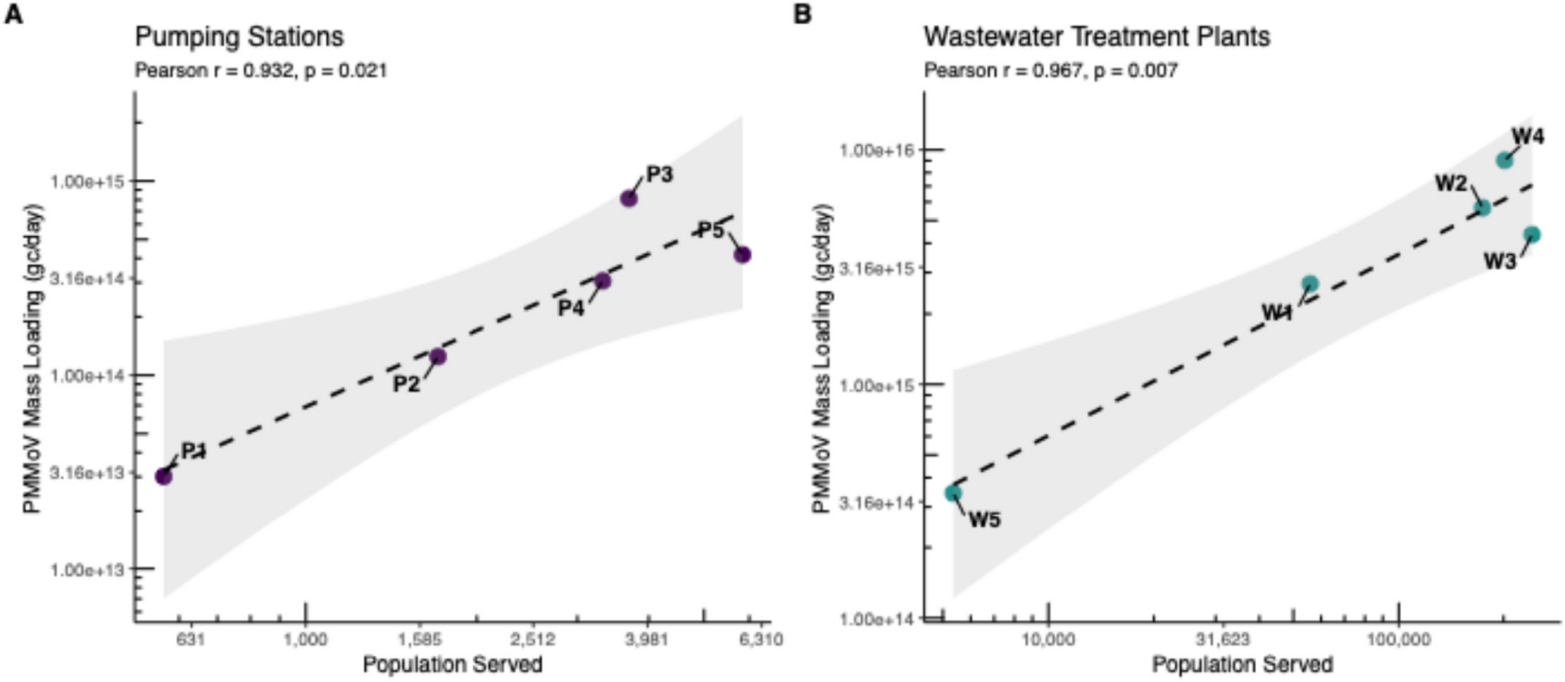
Relationship between PMMoV mass loading and estimated population served. Scatterplots showing PMMoV mass loading as a function of estimated population served for (A) pumping stations and (B) WWTPs. Lines indicate fitted regression models with gray areas indicating the 95% confidence intervals.

Catchment population size emerged as the dominant factor shaping wastewater dynamics. Flow rate was strongly and positively correlated with estimated population at pumping stations (*r* = 0.830, *p* < 0.05) and at WWTPs (*r* = 0.904, *p* < 0.05). Although population was not significantly correlated with PMMoV concentration, it was strongly correlated with PMMoV mass loading on a log–log scale (*r* = 0.932, *p* < 0.05). Similar relationships have been reported at WWTPs (*r* = 0.967, *p* < 0.05) (Figure 4).

Together, these findings indicate that although measured concentrations are shaped by local hydraulic conditions, the total viral mass entering the system increases predictably with the number of contributing residents. This relationship explains the strong variability observed at P1 (approximately 600 residents, *n* = 564), which serves a very small population and operates at low average flow. Although variability at sub‐1000 population scales could theoretically reflect intrinsic stochasticity in community shedding patterns, PMMoV is a dietary biomarker shed continuously and relatively uniformly by the population rather than an infection‐associated target subject to outbreak‐like dynamics. Consequently, substantial epidemiological fluctuations in PMMoV input are unlikely to be the primary driver of observed temporal variability, particularly in the absence of major shifts in population‐wide dietary habits during the study period. Instead, we attribute the elevated variability at P1 primarily to hydraulic and infrastructural factors: Limited mixing and extended wet‐well retention at this site allow wastewater to remain in the system for over an hour prior to pumping, conditions that can modify viral RNA profiles and magnify short‐term fluctuations. At the sub‐1000 resident scale, minimal water movement and low flows (0.014 MGD) contributed to “spiky” time series and elevated variability, whereas larger catchments exhibited more stable viral mass loading that were proportionally with population. Consequently, much of the apparent “noise” in smaller catchments reflects demographic and hydraulic constraints rather than environmental interference or epidemiological variability. The transition from WWTP‐scale monitoring to near‐source surveillance improved spatial resolution and reduced the distance between shedding population and sampling point (D'Aoust, Towhid, et al. 2021; Nguyen Quoc et al. 2022; Saingam et al. 2023; Kumar et al. 2023). However, our results demonstrated that very small catchments may fall below a practical lower limit for reliable surveillance, emphasizing the need to balance spatial resolution with hydraulic stability.

### 3.3 Surveillance of SARS‐CoV‐2, IAV, and RSV in Wastewater

Across all samples, SARS‐CoV‐2 RNA exhibited the highest detection frequency (85.5%), followed by RSV (28.4%) and IAV (28.6%). PMMoV was detected in all samples, confirming its ubiquitous presence as a fecal indicator throughout the study period. Mean concentrations were 5.22 ± 0.71 log_10_ gc/L for SARS‐CoV‐2, 4.17 ± 0.43 log_10_ gc/L for RSV, and 2.56 ± 1.05 log_10_ gc/L for IAV (Table S5). These patterns indicated sustained endemic circulation of SARS‐CoV‐2 throughout the study period, whereas RSV and IAV followed more episodic, seasonal dynamics. Spatial heterogeneity was most evident for SARS‐CoV‐2. Detection frequencies exceeded 90% at P2–P5 but were substantially lower at P1 (58.73%). A Kruskal–Wallis test confirmed significant spatial differences in SARS‐CoV‐2 concentrations (*χ* ^2^ = 43.59, *p* < 0.05), with post hoc analysis showing that P1 had significantly lower concentrations than all other sites. This pattern is consistent with the hydraulic and population constraints observed at P1 rather than true absence of infection.

In contrast, no significant spatial differences were detected for RSV (*χ* ^2^ = 6.71, *p* = 0.152) or IAV (*χ* ^2^ = 2.21, *p* = 0.698). Similar observations have been reported in longitudinal multi‐site studies, where seasonal respiratory viruses display synchronized regional waves once community transmission is established (Boehm et al. 2023; Daroch et al. 2025). The short, intense transmission windows of RSV and IAV likely mask localized heterogeneity that might otherwise be detectable during prolonged circulation.

Wastewater epidemic curves closely mirrored clinical hospitalization trends. RSV wastewater concentrations peaked on October 27, 2022 with 4.21 log_10_ gc/L, 2 days before the clinical peak on October 29, 2022. IAV peaked in wastewater on November 15, 2022 with 3.32 log_10_ gc/L, approximately 18 days prior to the clinical peak on December 3, 2022. SARS‐CoV‐2 peaked on January 5, 2023 with 6.38 log_10_ gc/L, 3 days before the clinical peak on January 8, 2023, consistent with previously reported lead times (D'Aoust, Graber, et al. 2021; Ahmed et al. 2021; Kumar et al. 2022; Kanchan et al. 2024).

### 3.4 Comparative Analysis of Wastewater Signals and Clinical Indicators

**FIGURE 5:**
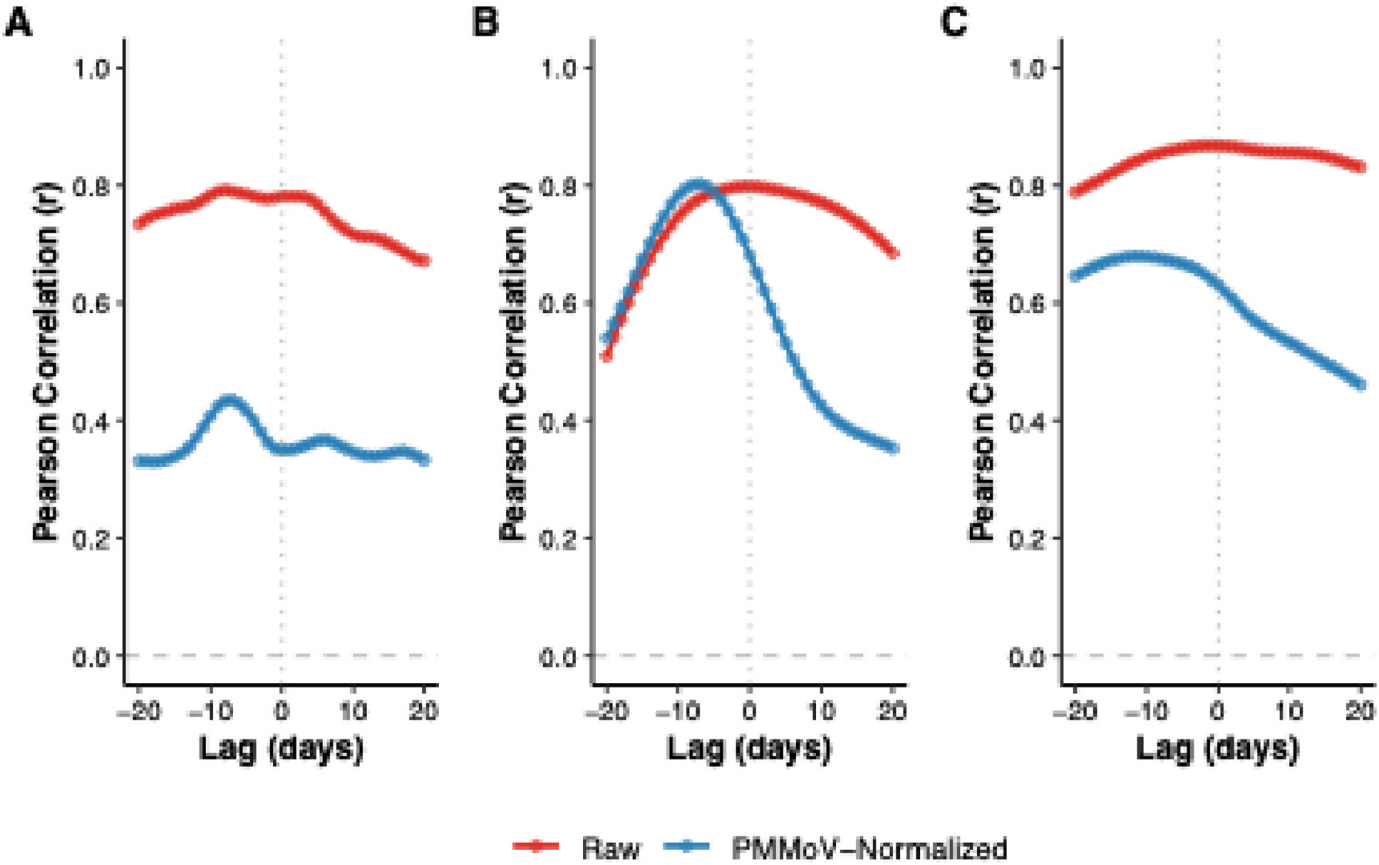
Lagged cross‐correlations between wastewater viral targets and clinical surveillance indicators. Lagged cross‐correlation plots showing Pearson correlation coefficients (*r*) as a function of time lag for (A) SARS‐CoV‐2, (B) influenza virus A (IAV), and (C) respiratory syncytial virus (RSV). Negative lags indicate that wastewater signals lead clinical indicators.

Time‐lagged cross‐correlation analyses demonstrated strong concordance between wastewater viral signals and clinical hospitalizations. For raw concentrations, RSV exhibited the highest correlation (*r* = 0.868, *p* < 0.05) at a 1‐day lead, IAV showed a maximum correlation of *r* = 0.798 (*p* < 0.05) at 0 days, and SARS‐CoV‐2 peaked at *r* = 0.792 (*p* < 0.05) with an 8‐day lead (Figure 5). The lead times identified in this study fell within ranges reported in previous WBE studies (D'Aoust, Graber, et al. 2021; Ahmed et al. 2021; Kumar et al. 2022; Kanchan et al. 2024). Variation in predictive windows was linked to virus‐specific biological kinetics. Pre‐Omicron SARS‐CoV‐2 variants exhibited a median incubation period of approximately 5 days, whereas Omicron variants showed shorter incubation periods of 2–3 days (CDC 2023). RSV typically has an incubation period of 3–5 days, with shedding lasting up to 8 days or as long as 3 weeks in vulnerable populations (Eiland 2009; Bennett et al. 2019), whereas influenza generally exhibits a shorter shedding duration of less than 5 days (Carrat et al. 2008).

Since viral shedding often begins during the incubation phase, wastewater signals frequently increase prior to symptom onset, providing an intrinsic early‐warning advantage over traditional clinical surveillance (Bertels et al. 2022). This advantage is further enhanced by delays in clinical testing and reporting, particularly with the widespread adoption of at‐home testing, making wastewater surveillance a more equitable proxy for community transmission (Maree et al. 2025; Wannigama et al. 2024). However, lead time is also influenced by infrastructural and environmental processes. Sewer holding and transit time and the 24‐h composite sampling event, all together introduce baseline technical delays (Bertels et al. 2022). In addition, temperature‐driven RNA decay and adsorption to sewer biofilms can shift the observed signal timing (Zhang et al. 2023; Li, Ahmed, et al. 2023; Medina et al. 2022). Methodological factors, including concentration approaches and the presence of degrading RNases during transport, further contribute to variability in apparent lead times (Sanchez‐Quete et al. 2024).

The effect of PMMoV normalization varied between viral targets. Normalization slightly increased IAV correlation (*r* = 0.802) and extended its lead time to 7 days but reduced correlations for SARS‐CoV‐2 (*r* = 0.435) and RSV (*r* = 0.679). These results indicate that PMMoV normalization did not universally improve predictive performance and may dampen true shedding signals at near‐source scale (Table 2), a finding consistent with literature reporting inconsistent benefits across different catchments (Maal‐Bared et al. 2023; Nagarkar et al. 2022; Feng et al. 2021; Greenwald et al. 2021).

**TABLE 2:** Maximum cross‐correlation coefficients and corresponding lead times for raw and PMMoV‐normalized viral data.

Targets	Normalization	Max Pearson's *r*	Estimated lead day	*p*
SARS‐CoV‐2	Normalized	0.435	−7	< 0.001
	Raw	0.792	−8	< 0.001
IAV	Normalized	0.802	−7	< 0.001
	Raw	0.798	0	< 0.001
RSV	Normalized	0.679	−11	< 0.001
	Raw	0.868	−1	< 0.001

Although some studies reported reduced noise in specific sewersheds (Nagarkar et al. 2022; D'Aoust, Mercier, et al. 2021), others demonstrated meaningful improvement in a small subset of systems, often excluding large or combined sewersheds (Maal‐Bared et al. 2023). The absence of a consistent relationship between PMMoV and population size suggested that observed benefits may reflect correction for sample processing variability, matrix effects, or storage losses rather than true population normalization. Consequently, although PMMoV remains widely used, its application should be considered on a case‐by‐case basis, and additional research is needed to identify alternative or complementary normalization markers that are specific to local or regional conditions.

## 4 Conclusion and Limitation

This study demonstrated that near‐source, community‐scale wastewater surveillance can effectively capture localized dynamics of SARS‐CoV‐2, IAV, and RSV. Monitoring at five upstream pumping stations improved spatial resolution relative to administrative boundaries and provided a direct representation of transmission within defined sewershed catchment areas. Wastewater signals consistently preceded clinical hospitalizations, with lead times 0 day for IAV, 1 day for RSV, and 8 days for SARS‐CoV‐2, supporting the value of wastewater surveillance as an early indicator of community infection trends.

PMMoV normalization showed clear target‐specific performance. Normalization improved agreement between wastewater and clinical data for IAV but reduced correlations for SARS‐CoV‐2 and RSV, indicating that unnormalized concentrations may better reflect community shedding for some respiratory viruses at the neighborhood scale. PMMoV also exhibited consistent winter–spring seasonality across sites, demonstrating that it was not temporally constant. Comparison of upstream pumping stations with downstream WWTP influent revealed pronounced signal smoothing at larger spatial scales, whereas near‐source sites showed greater hydraulic variability. Together, these findings indicate that near‐source surveillance offers higher geographic resolution, but normalization approaches must be selected based on viral target and catchment characteristics.

Several limitations should be acknowledged. The smallest catchment (P1; *n* = 564) exhibited highly variable time series, most likely driven by low flow and extended wet‐well retention, suggesting a practical lower limit for reliable near‐source monitoring. In addition, the drivers of PMMoV seasonality remain uncertain and may include dietary patterns or regional differences in food consumption. Future studies should evaluate alternative or complementary population biomarkers and further examine how site‐specific hydraulics and wastewater chemistry influence RNA persistence, with the goal of improving the reliability of community‐scale wastewater surveillance.

## Author Contributions

**Yuzhu Mao:** conceptualization, methodology, formal analysis, investigation, data curation, writing – original draft, writing – review and editing, visualization. **Chunfu Liu:** investigation, resources, writing – review and editing. **Rushan Abayagunawardena:** investigation. **Kosala Desilva:** investigation. **Patricia Arcellana:** investigation and formal analysis. **Birthe V. Kjellerup:** conceptualization, investigation, methodology, writing – review and editing, visualization, supervision, project administration, funding acquisition.

## Conflicts of Interest

The authors declare no conflicts of interest.

## Supporting information


**Figure S1:** Seasonal distributions of PMMoV concentrations at individual near‐source sampling sites. Seasonal boxplots of PMMoV concentrations for (A) Site P2, (B) Site P3, (C) Site P4, and (D) Site P5.
**Figure S2:** Temporal trends in wastewater flow rates at individual near‐source sampling sites. (A) Time series of daily wastewater flow rates for each site. (B) Boxplots summarizing the distribution of flow rates across sites.
**Table S1:** cDNA synthesis protocol used for viral RNA quantification.
**Table S2:** Primer and probe sequences used for quantification of PMMoV and viral targets.
**Table** S3. qPCR assay efficiency and coefficient of determination (*R* ^2^) for each target.
**Table S4:** Characteristics of the five wastewater treatment plants.
**Table S5:** Summary of viral concentrations and variability across study sites.

## Data Availability

All the data and code used in the research are available upon request.
