# Supplementary material for: Near‐Source Wastewater Surveillance of SARS‐CoV‐2, Influenza A, and Respiratory Syncytial Virus With Spatiotemporal Evaluation of Pepper Mild Mottle Virus as a Fecal Indicator
Source: Water Environ Res. 2026 May 15;98:e70414. doi: 10.1002/wer.70414 (PMC13178204; doi:10.1002/wer.70414)
Supplement: Supplementary file 1 — Figure S1: Seasonal distributions of PMMoV concentrations at individual near‐source sampling sites. Seasonal boxplots of PMMoV concentrations for (A) Site P2, (B) Site P3, (C) Site P4, and (D) Site P5. Figure S2: Temporal trends in wastewater flow rates at individual near‐source sampling sites. (A) Time series of daily wastewater flow rates for each site. (B) Boxplots summarizing the distribution of flow rates across sites. Table S1: cDNA synthesis protocol used for viral RNA quantification. Table S2: Primer and probe sequences used for quantification of PMMoV and viral targets. Table S3. qPCR assay efficiency and coefficient of determination (R 2) for each target. Table S4: Characteristics of the five wastewater treatment plants. Table S5: Summary of viral concentrations and variability across study sites. [file WER-98-e70414-s001.docx]

**Figure S1. Seasonal distributions of PMMoV concentrations at individual near-source sampling sites.** Seasonal boxplots of PMMoV concentrations for (A) Site P2, (B) Site P3, (C) Site P4, and (D) Site P5.

**Figure S2. Temporal trends in wastewater flow rates at individual near-source sampling sites.** (A) Time series of daily wastewater flow rates for each site. (B) Boxplots summarizing the distribution of flow rates across sites.

**Table S1. cDNA synthesis protocol used for viral RNA quantification.**

| Reaction mix 1: | vol. /50 µL, µL |
| --- | --- |
| H2O (RNase-free), µL | 6.5 |
| dNTP mix (10mM) | 2.5 |
| Random Hexamer (50 µM) | 3 |
| Sample RNA | 12 |
| Total vol | 24 |
| Reaction mix 1 underwent denaturation at 70°C for 5 minutes, followed by a 5-minute cooling phase at 4°C | |
| Reaction mix 2: | vol. /50 µL, µL |
| RNase Inhibitor (20 U/µl) | 1 |
| 5X ProtoScript II Buffer | 10 |
| Protoscript II RT (200U/µl) | 2.5 |
| 0.1M DTT | 5 |
| H2O (RNase-free) | 7.5 |
| Total vol | 26 |
| Reaction mix 2 was added, incubated at 42°C for 1 hr, followed by enzyme inactivation at 70°C for 20 min, 4°C for 5 min, and then stored at -20°C | |

**Table S2. Primer and probe sequences used for quantification of PMMoV and viral targets.**

| **Assay** | **Description** | **Oligonucleotide Sequence (5’>3’)** | **Cycle condition** | **product length, bp** | **Reference** |
| --- | --- | --- | --- | --- | --- |
| **PMMoV** | Forward | GAGTGGTTTGACCTTAACGTTTGA | 95℃ for 3mins, 40 cycles of 95℃ for 10s, 55℃ for 30s. | 68 | (Lee et al., 2018) |
|  | Reverse | TTGTCGGTTGCAATGCAAGT |  |  |  |
|  | FAM | CCTACCGAAGCAAATG |  |  |  |
| **N1** | Forward | GACCCCAAAATCAGCGAAAT | 95℃ for 2mins, 45 cycles of 95℃ for 3s, 55℃ for 30s. | 72 | (Vogels et al., 2020) |
|  | Reverse | TCTGGTTACTGCCAGTTGAATCTG |  |  |  |
|  | FAM | ACCCCGCATTACGTTTGGTGGACC |  |  |  |
| **InfA** | Forward | CAAGACCAATCYTGTCACCTCTGAC | 95℃ for 2mins, 45 cycles of 95℃ for 15s, 55℃ for 30s. | 106 | (Mk et al., 2022; Shu et al., 2021) |
|  | Reverse | GCATTYTGGACA AAV CGT CTA CG |  |  |  |
|  | FAM | TGC AGT CCT CGC TCA CTG GGC ACG |  |  |  |
| **RSV** | Forward | GGCAAATATGGAAACATACGTGAA | 95℃ for 2mins, 45 cycles of 95℃ for 3s, 55℃ for 30s. | 117 | (Fry et al., 2010; Williams et al., 2023) |
|  | Reverse | GGCACCCATATTGTWAGTGATG |  |  |  |
|  | FAM | GCTGTGTATGTGGAGCCYTCGTGAAG |  |  |  |

**Table S3. qPCR assay efficiency and coefficient of determination (R²) for each target.**

| Targets | Efficiency | Slope | y intercept | R-square |
| --- | --- | --- | --- | --- |
| **PMMoV** | 101.85 | -3.278 | 40.726 | 0.999 |
| **N1** | 106.59 | -3.174 | 37.548 | 0.989 |
| **IAV** | 90.28 | -3.579 | 35.072 | 0.999 |
| **RSV** | 95.68 | -3.430 | 38.385 | 0.990 |

**Table S4. Characteristics of the five wastewater treatment plants.**

| WWTPs | Estimated 2023 Population | Service area (square miles) | Projected Flow (MGD) | Note |
| --- | --- | --- | --- | --- |
| W1 | 55,848 | 13.9 | 6.57 | Covered hospital |
| W2 | 174,257 | 102.4 | 25.25 | Covered hospital |
| W3 | 241,316 | 126.7 | 22.25 | Covered hospital |
| W4 | 201,694 | 46.9 | 16.18 | Covered hospital |
| W5 | 5,354 | 4.88 | 0.88 | / |

**Table S5. Summary of viral concentrations and variability across study sites.**

| Target | Site | Mean ± SD (log_10_ gc/L) | CV (%) | Raw Mean (gc/L) | Raw SD (gc/L) |
| --- | --- | --- | --- | --- | --- |
| PMMoV | P1 | 8.43 ± 0.59 | 7.1% | 5.75 × 10^8^ | 8.47 × 10^8^ |
|  | P2 | 7.89 ± 0.88 | 11.1% | 3.16 × 10^8^ | 5.00 × 10^8^ |
|  | P3 | 7.75 ± 0.84 | 10.8% | 2.73 × 10^8^ | 7.04 × 10^8^ |
|  | P4 | 7.88 ± 0.83 | 10.6% | 3.28 × 10^8^ | 7.47 × 10^8^ |
|  | P5 | 7.67 ± 0.72 | 9.4% | 1.66 × 10^8^ | 3.44 × 10^8^ |
|  | W1 | 7.81 ± 0.48 | 6.2% | 1.08 × 10^8^ | 1.14 × 10^8^ |
|  | W2 | 7.56 ± 0.59 | 7.8% | 5.92 × 10^7^ | 4.67 × 10^7^ |
|  | W3 | 7.48 ± 0.49 | 6.6% | 5.17 × 10^7^ | 5.64 × 10^7^ |
|  | W4 | 7.95 ± 0.54 | 6.8% | 1.47 × 10^8^ | 1.26 × 10^8^ |
|  | W5 | 7.75 ± 0.67 | 8.6% | 1.03 × 10^8^ | 9.93 × 10^7^ |
| SARS-CoV-2 | P1 | 4.88 ± 0.67 | 13.7% | 2.15 × 10^5^ | 3.58 × 10^5^ |
|  | P2 | 5.34 ± 0.82 | 15.3% | 8.03 × 10^5^ | 1.18 × 10^6^ |
|  | P3 | 5.17 ± 0.63 | 12.2% | 3.88 × 10^5^ | 6.57 × 10^5^ |
|  | P4 | 5.47 ± 0.72 | 13.2% | 9.21 × 10^5^ | 1.45 × 10^6^ |
|  | P5 | 5.14 ± 0.58 | 11.3% | 2.81 × 10^5^ | 3.37 × 10^5^ |
| RSV | P1 | 4.25 ± 0.62 | 14.5% | 6.33 × 10^4^ | 1.52 × 10^5^ |
|  | P2 | 4.15 ± 0.36 | 8.7% | 2.10 × 10^4^ | 2.71 × 10^4^ |
|  | P3 | 4.06 ± 0.27 | 6.7% | 1.37 × 10^4^ | 9.19 × 10^3^ |
|  | P4 | 4.16 ± 0.37 | 8.9% | 2.00 × 10^4^ | 1.73 × 10^4^ |
|  | P5 | 4.27 ± 0.55 | 12.9% | 4.67 × 10^4^ | 9.39 × 10^4^ |
| IAV | P1 | 3.16 ± 1.48 | 46.7% | 3.88 × 10^4^ | 8.52 × 10^4^ |
|  | P2 | 2.38 ± 0.85 | 35.5% | 9.46 × 10^2^ | 1.44 × 10^3^ |
|  | P3 | 2.49 ± 0.90 | 36.0% | 1.53 × 10^3^ | 2.43 × 10^3^ |
|  | P4 | 2.34 ± 0.94 | 40.1% | 1.24 × 10^3^ | 1.95 × 10^3^ |
|  | P5 | 2.49 ± 0.88 | 35.3% | 1.26 × 10^3^ | 1.89 × 10^3^ |

**Reference**

Fry, A.M., Chittaganpitch, M., Baggett, H.C., Peret, T.C.T., Dare, R.K., Sawatwong, P., Thamthitiwat, S., Areerat, P., Sanasuttipun, W., Fischer, J., Maloney, S.A., Erdman, D.D., Olsen, S.J., 2010. The burden of hospitalized lower respiratory tract infection due to respiratory syncytial virus in rural Thailand. PLoS One 5, e15098. https://doi.org/10.1371/journal.pone.0015098

Lee, H.-W., Lee, H.-M., Yoon, S.-R., Kim, S.H., Ha, J.-H., 2018. Pretreatment with propidium monoazide/sodium lauroyl sarcosinate improves discrimination of infectious waterborne virus by RT-qPCR combined with magnetic separation. Environmental Pollution 233, 306–314. https://doi.org/10.1016/j.envpol.2017.10.081

Mk, W., D, D., Km, B., M, A., L, M., B, H., P, A., As, L., Wj, F., E, B., Ce, H., Et, M., Bj, W., Ab, B., Kr, W., 2022. Wastewater-based detection of two influenza outbreaks. https://doi.org/10.1101/2022.02.15.22271027

Shu, B., Kirby, M.K., Davis, W.G., Warnes, C., Liddell, J., Liu, J., Wu, K.-H., Hassell, N., Benitez, A.J., Wilson, M.M., Keller, M.W., Rambo-Martin, B.L., Camara, Y., Winter, J., Kondor, R.J., Zhou, B., Spies, S., Rose, L.E., Winchell, J.M., Limbago, B.M., Wentworth, D.E., Barnes, J.R., 2021. Multiplex Real-Time Reverse Transcription PCR for Influenza A Virus, Influenza B Virus, and Severe Acute Respiratory Syndrome Coronavirus 2. Emerg Infect Dis 27, 1821–1830. https://doi.org/10.3201/eid2707.210462

Vogels, C.B.F., Brito, A.F., Wyllie, A.L., Fauver, J.R., Ott, I.M., Kalinich, C.C., Petrone, M.E., Casanovas-Massana, A., Catherine Muenker, M., Moore, A.J., Klein, J., Lu, P., Lu-Culligan, A., Jiang, X., Kim, D.J., Kudo, E., Mao, T., Moriyama, M., Oh, J.E., Park, A., Silva, J., Song, E., Takahashi, T., Taura, M., Tokuyama, M., Venkataraman, A., Weizman, O.-E., Wong, P., Yang, Y., Cheemarla, N.R., White, E.B., Lapidus, S., Earnest, R., Geng, B., Vijayakumar, P., Odio, C., Fournier, J., Bermejo, S., Farhadian, S., Dela Cruz, C.S., Iwasaki, A., Ko, A.I., Landry, M.L., Foxman, E.F., Grubaugh, N.D., 2020. Analytical sensitivity and efficiency comparisons of SARS-CoV-2 RT–qPCR primer–probe sets. Nat Microbiol 5, 1299–1305. https://doi.org/10.1038/s41564-020-0761-6

Williams, T., Jackson, S., Barr, I., Bi, S., Bhiman, J., Ellis, J., von Gottberg, A., Lindstrom, S., Peret, T., Rughooputh, S., Viegas, M., Hirve, S., Zambon, M., Zhang, W., Group, W.R.S., 2023. Results from the second WHO external quality assessment for the molecular detection of respiratory syncytial virus, 2019–2020. Influenza and Other Respiratory Viruses 17, e13073. https://doi.org/10.1111/irv.13073
